# Integrating fast and slow processes is essential for simulating human–freshwater interactions

**DOI:** 10.1007/s13280-018-1136-6

**Published:** 2018-12-19

**Authors:** Nicole K. Ward, Leah Fitchett, Julia A. Hart, Lele Shu, Jemma Stachelek, Weizhe Weng, Yu Zhang, Hilary Dugan, Amy Hetherington, Kevin Boyle, Cayelan C. Carey, Kelly M. Cobourn, Paul C. Hanson, Armen R. Kemanian, Michael G. Sorice, Kathleen C. Weathers

**Affiliations:** 1 Department of Biological Sciences, Virginia Tech, 926 West Campus Drive, Blacksburg, VA 24061 USA; 2 Department of Forest Resources and Environmental Conservation, Virginia Tech, 310 West Campus Drive, Blacksburg, VA 24061 USA; 3 Center for Limnology, University of Wisconsin - Madison, 680 N Park Street, Madison, WI 53706 USA; 4 Department of Civil and Environmental Engineering, The Pennsylvania State University, 212 East College Avenue, University Park, PA 16802 USA; 5 Department of Land, Air and Water Resource, University of California, Davis, 223 Hoagland Hall, Davis, CA 95616 USA; 6 Department of Fisheries and Wildlife, Michigan State University, 480 Wilson Road, East Lansing, MI 48824 USA; 7 Department of Agricultural and Applied Economics, Virginia Tech, 250 Drillfield Drive, Blacksburg, VA 24061 USA; 8 Nicholas School of the Environment, Duke University, 9 Circuit Drive, Durham, NC 27708 USA; 9 Department of Plant Sciences, The Pennsylvania State University, 116 ASI Building, University Park, PA 16802 USA; 10 Cary Institute of Ecosystem Studies, 2801 Sharon Turnpike, Millbrook, NY 12545 USA

**Keywords:** Coupled human–natural systems, Coupled modeling, Decision-making, Feedbacks, Water resources

## Abstract

Integrated modeling is a critical tool to evaluate the behavior of coupled human–freshwater systems. However, models that do not consider both fast and slow processes may not accurately reflect the feedbacks that define complex systems. We evaluated current coupled human–freshwater system modeling approaches in the literature with a focus on categorizing feedback loops as including economic and/or socio-cultural processes and identifying the simulation of fast and slow processes in human and biophysical systems. Fast human and fast biophysical processes are well represented in the literature, but very few studies incorporate slow human and slow biophysical system processes. Challenges in simulating coupled human–freshwater systems can be overcome by quantifying various monetary and non-monetary ecosystem values and by using data aggregation techniques. Studies that incorporate both fast *and* slow processes have the potential to improve complex system understanding and inform more sustainable decision-making that targets effective leverage points for system change.

**Electronic supplementary material:**

The online version of this article (10.1007/s13280-018-1136-6) contains supplementary material, which is available to authorized users.

## Introduction

Coupled natural and human system (CNHS) models are a critical tool to improve system understanding and to inform sustainable decision-making. Importantly, modeling enables experimentation and prediction in complex systems. Here, prediction is not intended to “see the future,” but rather to assist decision-makers in understanding potential futures or system trajectories based on today’s decisions (Srinivasan et al. 2017). For CNHS models to illustrate potential futures, they must simulate feedback loops, or the two-way interaction between human and biophysical system components (Box 1, Troy et al. 2015). Feedback loops are important to include in CNHS models because they may give rise to complex system behavior, such as non-linearities, time lags, and surprises (Hull et al. 2015). Environmental management decisions are often made in response to short-term dynamics, while ignoring complex system behavior over the long term, resulting in unintended consequences (Chapin et al. 2009). Thus, for CNHS models to effectively inform management decisions, it is critically important to incorporate the interaction of processes occurring over different time scales. We argue that incorporating *both* fast *and* slow feedbacks is critical for simulating CNHS dynamics in human–freshwater systems, yet this coupling is rarely represented in CNHS models.

**Table:** 

**Box 1** Glossary of terms used	
Coupled modeling	The linkage of multiple stand-alone disciplinary models to represent a coupled natural–human system (e.g., linking an economic decision-making model and a hydrologic model)
Feedback loop	The two-way interaction between human and biophysical system components in a coupled natural–human system (i.e., the human system affects the biophysical system *and* the biophysical system affects the human system). The diagram below depicts a feedback loop between biophysical and human systems in which each system may be characterized by interactions between potentially nested internal processes (e.g., between terrestrial and aquatic processes in the biophysical system and between economic and socio-cultural processes in the human system)
	
Model	Computer-based algorithm and simulation representing coupled natural–human system dynamics
Process	A series of reactions or operations that act on one or more variables. Processes may interact with other processes, creating linkages between variables (e.g., terrestrial and aquatic processes interact to drive changes in the variables that describe the biophysical state of the ecosystem, as depicted in the definition of a feedback loop, above)
Aquatic process	A series of reactions or operations acting on aquatic variables. Examples include eutrophication, phytoplankton productivity, decomposition, and nutrient cycling
Economic process	A series of reactions or operations that act on economic variables. Examples include market development and human decision-making in response to markets and policy incentives
Socio-cultural process	A series of reactions or operations that act on social, cultural, and institutional variables. Examples include preference formation, cultural and institutional change, and human decision-making in response to shared values or behavioral norms
Terrestrial process	A series of reactions or operations acting on terrestrial variables. Examples include crop nitrogen uptake, nitrogen leaching, decomposition, and nutrient cycling
Variable	A metric of the state of a system, e.g., soil nitrogen, crop price, behavioral norms, soil organic matter (Fig. 1); Variables may be either fast or slow, as depicted in the diagram below
	
Fast variable	Variables changed through fast processes acting over a relatively short time frame, such as days to years (e.g., crop yield and crop price, Fig. 1)
Slow variable	Variables that remain relatively constant over a short time frame, but may change through slow processes acting over a relatively long time frame, such as decades to centuries (e.g., soil organic matter and behavioral norms, Fig. 1)

**Fig. 1:**
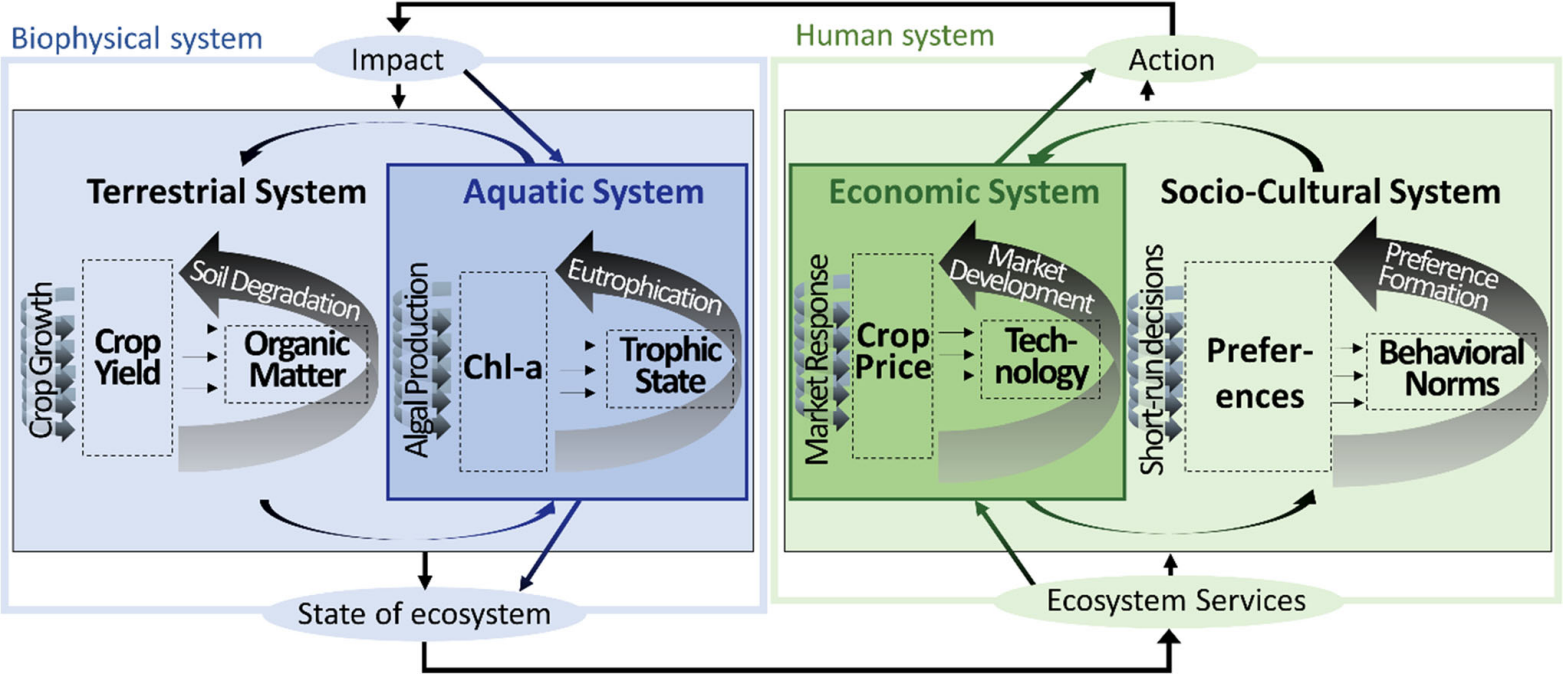
Examples of fast and slow variable and process interactions in terrestrial, aquatic, economic, and socio-cultural subsystems within a CNHS. Within biophysical systems, aquatic subsystem processes and variables operate within the context of the terrestrial subsystems. Similarly, within human systems, economic subsystem processes and variables operate within the context of the socio-cultural subsystem. Within each subsystem, fast processes and variables, such as algal production (process) and chlorophyll-a (chl-a; variable) change within the context of slow processes and variables, such as eutrophication (process) and trophic state (variable). Fast variables (e.g., crop yield) may contribute to abrupt change in slow variables (e.g., soil organic matter) if a threshold is crossed

Incorporating feedback loops that operate over short and long time scales into system models will improve the simulation of CNHS dynamics. A feedback loop occurs when a change in one part of the system elicits a response elsewhere in the system, causing further change that compounds or mediates the original change (Hull et al. 2015). Feedback loops in CNHS are made up of “fast” processes occurring over days and years and “slow” processes occurring over decades and centuries. Fast and slow processes alter fast and slow state variables, respectively (Box 1 and Fig. 1, see discussion of fast and slow variables in CNHS in Chapin et al. 2009). Fast state variables include, for example, water clarity, algal concentration, or crop yield in biophysical systems, and land management or crop price in human systems. Since fast processes operate on a time scale of days to years, fast variables often change from day to day or year to year. Fast processes occur within the context of slow processes, such as ecosystem regime shifts in biophysical systems or the emergence of new markets or cultural change in human systems (Fig. 1). Slow state variables include, for example, lake trophic state in biophysical systems and behavioral norms in human systems (Fig. 1). Slow variables are often stable from year to year, but may change over the course of decades or centuries. The change in a slow variable may occur incrementally over time or abruptly at threshold points (Chapin et al. 2009).

Environmental policy and management decisions are often short-term “quick-fixes” (Stroh 2015) made in response to fast variables that ignore the non-linearities and time lags inherent in complex systems (Levin et al. 2012) and cause unintended consequences (Chapin et al. 2009). Such quick-fix management decisions are among the least effective tools to initiate change in a system (Meadows 1999; Abson et al. 2017). In contrast to quick-fixes, effective policy and management decisions over the long term initiate deeper systemic change by considering overall system dynamics, including the interaction of fast and slow variables (Meadows 1999; Matson et al. 2016). Thus, for CNHS models to effectively inform management decisions, they must incorporate the interaction of variables changing over different time scales within system feedback loops. We argue that incorporating *both* fast *and* slow processes is critical for simulating CNHS dynamics in human–freshwater systems, yet this coupling is rarely represented in CNHS models.

We examined the current state of the scientific literature to determine whether and how fast and slow processes are represented in coupled models of human–freshwater systems. We found that the representation of slow processes is a rarity in coupled human–freshwater modeling, and that feedback loops with slow human system processes are most often simulated with socio-cultural modeling approaches. We present strategies to improve the representation of feedback loops in models of freshwater CNHS and highlight novel insights gleaned from studies that incorporate both fast and slow processes.

## Literature review

### Methods

There are many different types of modeling in CNHS. Here, we focus on coupled component modeling (or “coupled modeling”) in human–freshwater systems. Coupled modeling is the linkage of multiple (two or more) stand-along disciplinary models to represent a CNHS, for example, linking an economic decision-making model and a hydrologic model (Box 1). Coupled modeling maintains disciplinary rigor and enables the representation of processes at different hierarchical scales (Kelly et al. 2013). Throughout the paper, we use “model” to refer to computer-based algorithm and simulation models (Box 1). We chose to focus on human–freshwater systems as exemplary CNHS due to strong interdependencies in these systems (Cobourn et al. 2018). For example, freshwaters (lakes, rivers, wetlands, and groundwater) provide high-value ecosystem services (e.g., MEA 2005; de Groot et al. 2012), yet the services they provide are often affected by human activities such as land-use practices (Carpenter et al. 2011).

We reviewed the scientific literature related to coupled modeling in human–freshwater systems for all papers available on abstract index databases on the topic before 15 August 2017. We identified 601 peer-reviewed papers through keyword searches in Web of Science™ Core Collection by Thomson Reuters, SocINDEX by EBSCO host, and Water Resources Abstracts by ProQuest. We also included relevant citations identified in references cited within the 601 papers. We screened all abstracts and narrowed our pool of modeling papers to those that focused on freshwater systems and those that used a coupled modeling approach combining at least one process-based biophysical system model and one human system model. We focused on process-based biophysical models due to the need to represent complex biophysical interactions (Kelly et al. 2013) and because of the challenges of coupling highly detailed, spatially distributed biophysical models with human system models. For a detailed description of literature review methods and the keyword search terms used, see supplemental material (S1).

**Fig. 2:**
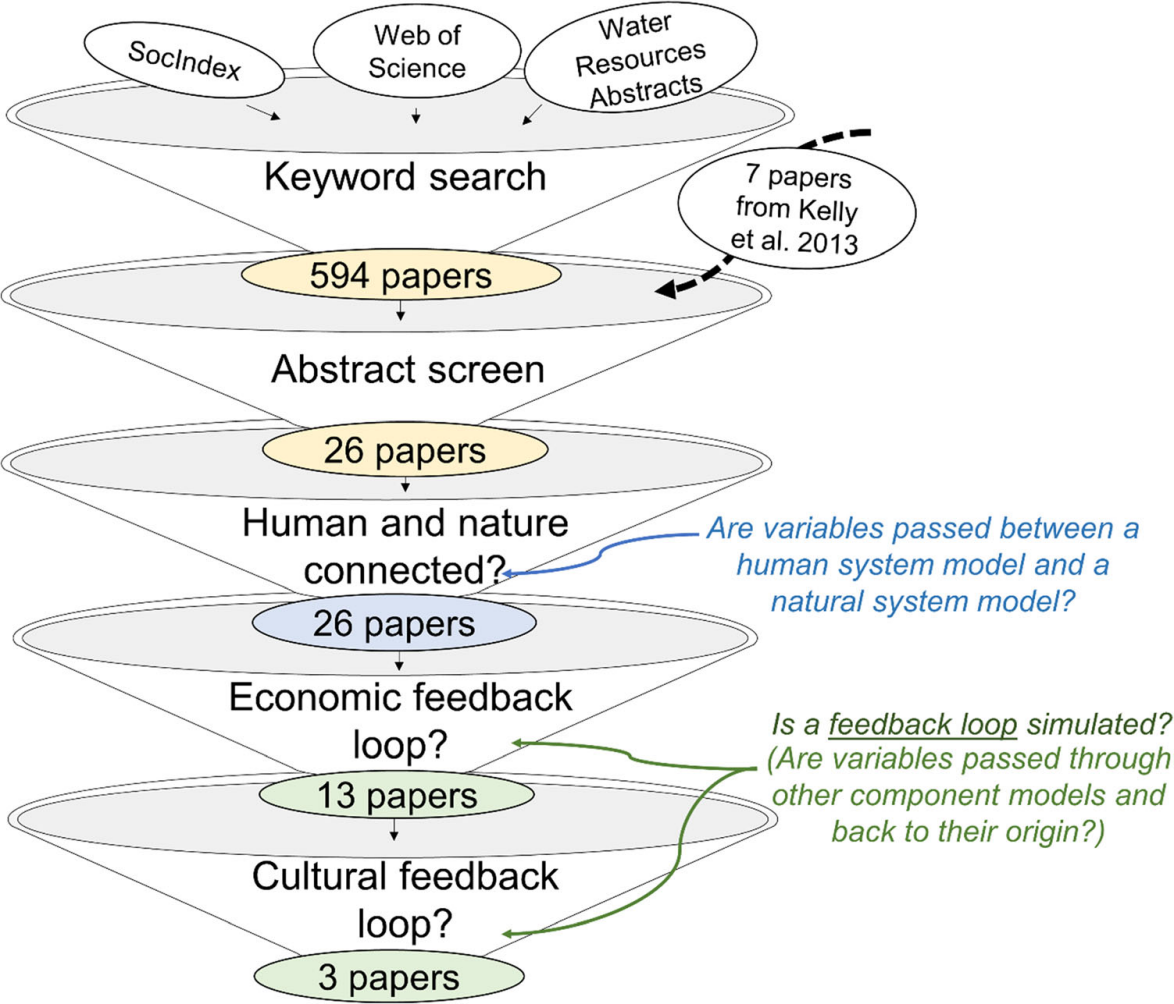
Coupled modeling literature review search and categorization methods, see S1 for full methods description including identification of papers from Kelly et al. (2013). All 26 selected studies passed variables from human to biophysical system models; 13 of the 26 studies simulated a feedback loop from the biophysical system to the economic subsystem (and back into the biophysical system); 3 of the 26 studies simulated a feedback loop from the biophysical system to both the economic and cultural subsystems (and back into the biophysical system)

**Fig. 3:**
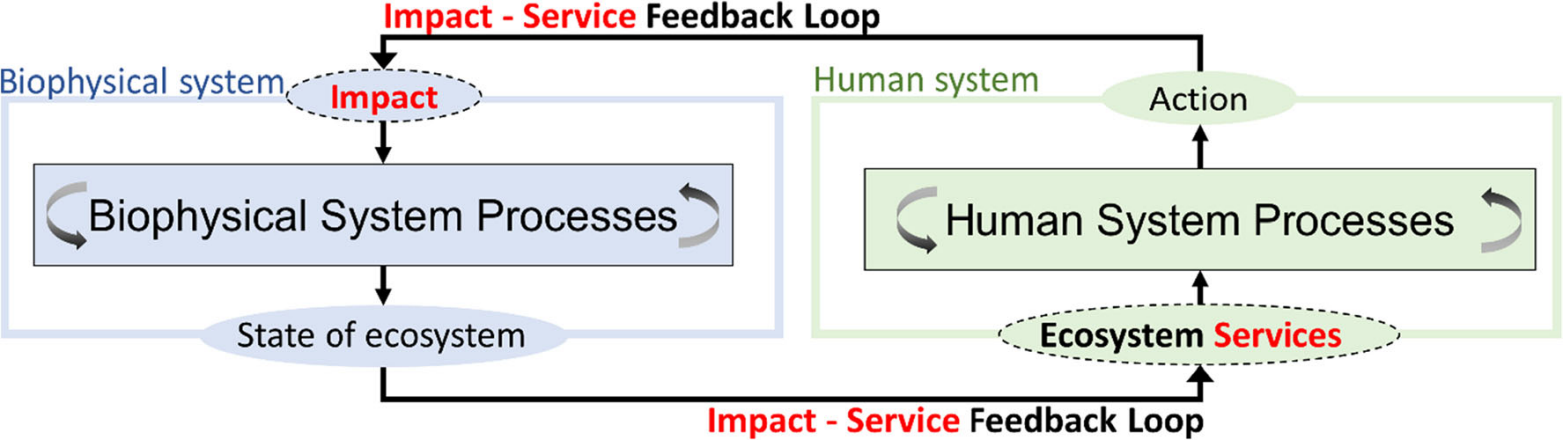
Impact-service feedback loop, modified from Collins et al. (2011). Human actions *impact* the natural system through pulse and press disturbances, and the state of the ecosystem determines the *ecosystem services* that are available to the human system. Therefore, human and natural system models are connected through *human impact* and *ecosystem services* via the impact-service feedback loop

In total, we identified 26 papers meeting our selection criteria (Fig. 2). To compare papers across different model platforms and conceptual representations, we mapped modeling approaches on a generalizable “impact-service feedback” conceptual diagram (Fig. 3, adapted from Collins et al. (2011)). The impact-service conceptual model frames the connection from the human system to the biophysical system as “impacts” (e.g., water use, conservation practice), and the connection from the biophysical system to the human system as “services” provided (e.g., ecosystem services such as water availability and erosion control). The impact-service conceptual framework explicitly highlights the variables passed between human system models and biophysical systems models.

We classified these 26 papers based on the type of feedback represented and the types of processes simulated in the coupled model: no feedback loop, a feedback loop with economic processes, and a feedback loop with economic and socio-cultural processes (Box 1). We define an economic process as a series of reactions or operations that act on economic variables. Examples of economic processes include market development and human decision-making in response to markets and policy incentives (Box 1). We define a socio-cultural process as a series of reactions or operations that act on social, cultural, and institutional variables. Examples of socio-cultural processes include preference formation, cultural and institutional change, and human decision-making in response to shared values or behavioral norms (Box 1). Economic and socio-cultural processes interact to form human preferences and drive human decision-making. Additionally, we identified whether studies represented terrestrial and/or aquatic processes (see Box 1 for definitions) and identified whether each study simulated fast and/or slow processes in the human system and biophysical system.

### Results

Of the 26 studies, 13 were categorized as having no feedback loops (Table 1). Of these 13 studies without feedback loops, ten simulated the slow biophysical process of water supply change (e.g., groundwater depletion), and no papers simulated slow human processes (Table 1). These coupled models without feedback loops were composed of unidirectional impacts; for example, output variables from a human system model were used as input variables to a biophysical system model, but not back to the human system. These studies often used a suite of scenario simulations based on previous simulation outcomes (i.e., the model output at the end of the first simulation, such as water supply, was then used as input for the next scenario), creating a model-user mediated form of feedback loop. For example, Yaeger et al. (2014) ran a human system optimization model to determine land-use inputs for a hydrologic model. The hydrologic outcomes were then used as inputs to the human decision-making model in the next simulation. This is a “no feedback loop” example because the hydrologic outcomes are not automatically fed back to the human decision-making model within one simulation.

**Table 1:** Example feedback loop types, processes simulated, variables passed between models, and years of simulation for each of the studies we reviewed. Econ is an abbreviation for Economic processes; SC is an abbreviation for Socio-Cultural processes. Fast variables are italicized and slow variables are underlined

Feedback loop type	Processes simulated	Example variable(s) passed between models	Years simulated	Citation
No feedback loop	Econ + SC → Aquatic	*Land management (conservation practices)*	41	Daloğlu et al. (2014)
	Econ → Aquatic	*Land use (crop and fertilizer)*	15	Gandolfi et al. (2014)
	Econ → Aquatic	*Impervious surface area (ha)*	30	Hong et al. (2012)
	Aquatic → Econ	Water supply (m^3^); *Stream discharge (m*^*3*^*/s)*	NA	Kokkinos et al. (2014)
	Aquatic → Terrestrial → Econ	Groundwater (m); *Soil moisture (m*^*3*^*/m*^*3*^*); Crop yield (t/ha)*	50	Krol et al. (2001)
	Aquatic → Terrestrial → Econ	Groundwater (m); *Stream discharge (m*^*3*^*/s); Crop yield (t/ha)*	20	Magombeyi and Taigbenu (2011)
	Econ → Aquatic	*Water use (m*^*3*^*/month)*	40	Sato et al. (2009)
	Aquatic → Econ	*Water availability (m*^*3*^*/s)*	50	Skoulikaris et al. (2009)
	Econ → Aquatic	*Water demand (m*^*3*^*/yr);* Groundwater (m)	3–17	Varela-Ortega et al. (2011)
	Aquatic → Econ	*Concentration of algae (µg/L)*	30	van der Veeren and Lorenz (2002)
	Econ → Aquatic	*Land use (crop); Water quality (concentration of N);* Water supply (m^3^)	45	Yaeger et al. (2014)
	Aquatic → Econ	Water supply (m^3^); *Water availability (m*^*3*^*/s)*	14	Xiang and Jun (2009)
	Econ + SC → Terrestrial → Aquatic	*Landowner benefit; Land use (fertilizer rate); Runoff (mm);* Groundwater (m)	40	Zia et al. (2016)
Feedback loop with Econ processes	Econ → Terrestrial → Aquatic → Econ	*Total income (€); Land use (ha); Surface water availability (Hm*^*3*^*/yr);* Groundwater (Hm); *Water use (Hm*^*3*^*/yr)*	8	Cabello et al. (2015)
	Econ → Aquatic → Econ	*Water use (m*^*3*^*);* Groundwater (m^3^); *Water transport (km)*	4–20	Grundmann et al. (2012)
	Aquatic → Econ → Aquatic	*Stream discharge (m*^*3*^*/s);* Water shortage degree; *Water use (m*^*3*^*)*	50	Jia et al. (2009)
	Econ → Aquatic → Terrestrial → Econ	*Municipal income ($/yr);* Water supply (m^3^); *Crop yield (t/ha); Farm income ($/yr)*	100	Krol and Bronstert (2007)
	Econ → Terrestrial → Aquatic → Econ	*Crop management (crop type and fertilizer use); Crop yield (t/ha); Crop water demand (mm); Surface water availability (m*^*3*^*/10* *days)*	5	Letcher et al. (2006)
	Aquatic → Econ → Aquatic	*Land use (crop and management); Water quality (nitrogen conc.)*	12	Rutledge et al. (2008)
	Aquatic → Econ → Aquatic	*Water use (m*^*3*^*/yr);* Water supply (m); Technology adoption	40	Srinivasan (2015)
	Terrestrial → Econ → Aquatic → Terrestrial	*Crop yield (t/ha); Water demand (m*^*3*^*/day);* Water supply (m^3^); *Water availability (m*^*3*^*/day); Crop growth (leaf area index);* Fertile soil depth (m)	30	van Delden et al. (2007)
	Econ → Aquatic → Econ	*Land value ($/ha); Land cover (forested);* Groundwater (m)	5	Voinov et al. (2007)
	Aquatic → Econ → Aquatic	Water supply (*m*^*3*^*); Water availability (m*^*3*^*/yr); Water demand (m*^*3*^*/yr)*	18	Zeng et al. 2012
Feedback Loop with Econ and SC processes	Terrestrial → Aquatic → SC → Econ → Terrestrial	*Land cover (agriculture);* Groundwater (m^3^); *Water quality (cyanobacteria: µg/L);* Community sensitivity; *land clearing (ha/yr)*	100	Elshafei et al. (2014)
	Econ → Aquatic → SC → Econ	*Water demand (m*^*3*^*/yr); Water availability (m*^*3*^*/yr); pluralistic stakeholder preferences*	NA	Fedra (2007)
	Econ → Aquatic → SC → Econ	*Phosphorus load (kg/yr); Water clarity (# of clear water days); Environmental satisfaction (# of clear water days: desired);* Level of engagement (0–1)	50	Roy et al. (2011)

**Fig. 4:**
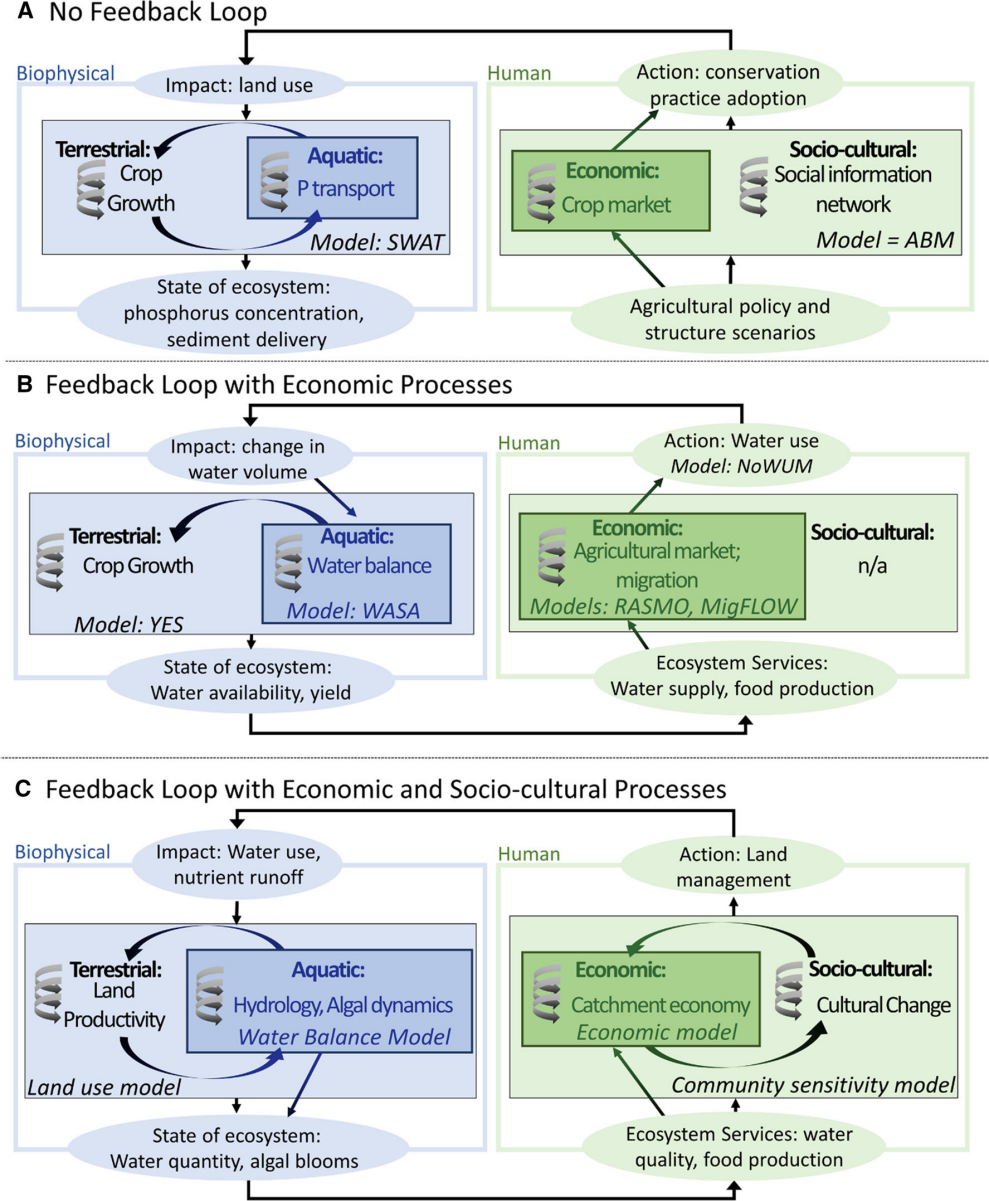
Example coupled modeling studies mapped onto the impact-service feedback loop. **a** No feedback loop is represented because no linkage is made from the biophysical system to the human system (Daloğlu et al. 2014); **b** A feedback loop is represented with the human system consisting exclusively of economic processes (Krol and Bronstert 2007); **c** A feedback loop is represented with the human system consisting of both economic and socio-cultural processes (Elshafei et al. 2014). Studies with or without a feedback loop differ in the biophysical and human process interactions that are represented. For example, **a** and **c** illustrate cases in which aquatic systems affect terrestrial systems and vice versa; in **b** the aquatic system influences the terrestrial system. Similarly, **a** illustrates a study that includes economic and socio-cultural processes but no interaction between them; **b** illustrates a study with only economic processes; and **c** illustrates a study with both economic and socio-cultural processes as well as their bi-directional interactions. The processes modeled as part of the biophysical system and human system shapes the nature of CNHS connections in the impact-service feedback loop

Alternatively, “no feedback loop” scenarios may be determined separately from baseline simulation outcomes. For example, Daloğlu et al. (2014) used scenarios to test the effect of different agricultural policies and non-operator (absentee) landowner involvement on water quality outcomes. They used an agent-based model (ABM) to simulate producer adoption of conservation practices under policy and non-operator involvement scenarios. Producer decisions were based on net farm income, land management preferences, and influence from neighbors. Output of the ABM (e.g., adoption of conservation practices) was converted to a land-use map, which was then used as input to the Soil and Water Assessment Tool (SWAT). Daloğlu et al. (2014) then used SWAT to assess the amount of sediment and phosphorus loss associated with each scenario (Fig. 4a). “No feedback loop” coupled models such as described in these two studies can increase understanding of the current state of the system and are appropriate for short time scale questions, but may be limited in application to longer time scales because they do not simulate feedback loops that give rise to potential complex system dynamics. However, applying different scenarios to models with no feedback loops can improve applicability of the results to longer time scales.

Twenty-eight percent (*n* = 10) of the papers simulated feedback loops with economic processes, but did not simulate socio-cultural processes. These coupled models passed variables both *from* a human decision-making model to a biophysical system model and from a biophysical system model *to* a human decision-making model. Of these ten studies, seven simulated the slow biophysical process of water supply change (e.g., groundwater depletion), one simulated the slow biophysical processes of soil fertility change, and one paper simulated a slow human system process (Table 1). Srinivasan (2015) simulated the slow human system process of technological innovation emergence and adoption of new technology as a result of market drivers. Srinivasan (2015) presents a simplified (“stylized”) coupled model, where distinct algorithms represent key biophysical system and human system variables (e.g., water quantity and price) based on a previously used coupled component model.

Krol and Bronstert (2007) provide an example of linking disciplinary models in the full impact-service feedback loop with economic human system processes. They used a water-balance model (WASA) and an agricultural yield model (YES) to represent the biophysical system. Outputs of water balance and yield were used to determine the ecosystem services of water supply and food production, which were then used as inputs to a water-use model (NoWUM) and a regional agricultural economy model (RASMO). Outputs from the economic model drove changes in population dynamics (MigFLOW), which were used as input to the water-use model. Finally, the water-use model outputs were used to drive human impact on the water-balance model, completing the impact-service feedback loop (Fig. 4b).

Only twelve percent (*n* = 3) of the papers simulated feedback loops with socio-cultural processes in addition to economic processes (Table 1). One paper simulated the slow biophysical system process of water supply change (Elshafei et al. 2014). Two of these three papers simulated slow human system processes (Roy et al. 2011; Elshafei et al. 2014). For example, Elshafei et al. (2014), simulated water availability, quality, and agricultural production (fast processes) using a water-balance model and a land-use model. Model outputs of ecosystem services (water quality, food production, etc.) then interacted with the slow process of cultural change and fast economic processes to affect the slow variable of “community sensitivity,” or the community’s perceived threat an environmental issue posed to their quality of life (Fig. 4c). Elshafei et al. (2014) found that when community sensitivity is high, the socio-cultural regime is enviro-centric, where environmental conservation measures are more likely to be employed. In this model application, the behavioral response to a given level of ecosystem services depends on the current state of the community sensitivity in the catchment.

**Fig. 5:**
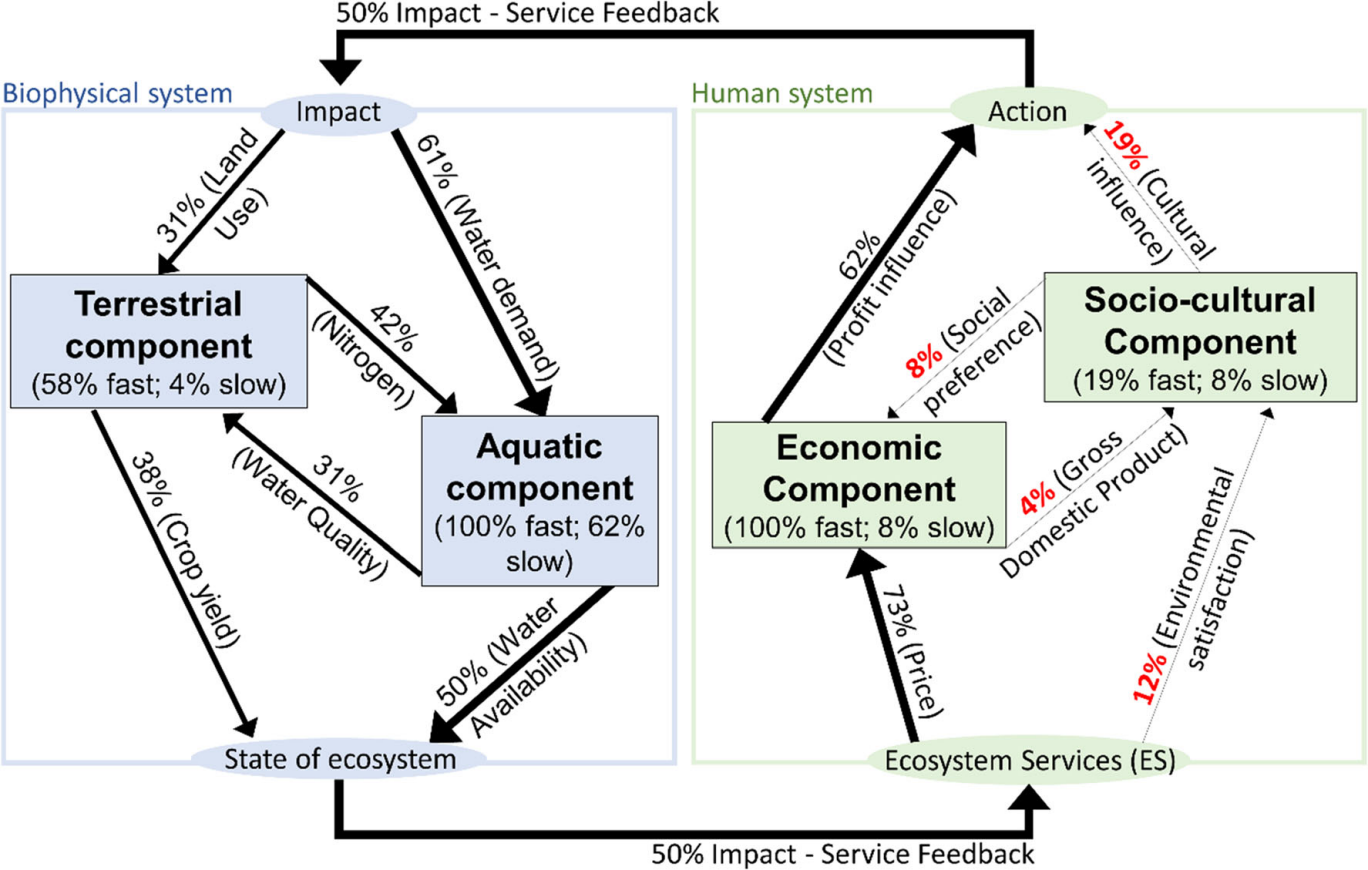
Percentage of all 26 papers that simulated a given component connection and variable type (fast or slow). Thicker lines indicate more commonly simulated linkages; all percentages below 20 are presented in red text to highlight the least commonly simulated linkages

Overall, 50% (*n* = 13) of the papers we reviewed simulated the full impact-service feedback loop while only three papers included socio-cultural processes (Fedra 2007; Roy et al. 2011; Elshafei et al. 2014) and only three papers included slow human system processes (Roy et al. 2011; Elshafei et al. 2014; Srinivasan 2015) (Table 1, Fig. 5). All of the 26 papers simulated fast human and biophysical system processes, with water quantity and price as the most common variable linkages. Within the biophysical system, 62% (*n* = 16) papers simulated slow freshwater processes, all of which were focused on surface and groundwater depletion. Only one paper simulated a slow terrestrial system process, changes to soil fertility (van Delden et al. 2007).

The rarity of CNHS models that include feedback loops with both economic and socio-cultural processes is likely a result of a less-established history of disciplinary collaboration between biophysical and socio-cultural researchers and more disparate disciplinary methodologies. Feedback loops with economic processes are likely more common because biophysical scientists and economists rely on relatively similar quantitative methodologies to represent system processes (Schlüter et al. 2017). For example, decision-making theories in economics are often represented using mathematical formulas, such as ordinary differential equations, which are similar to biophysical process-based model algorithms (Schlüter et al. 2017). These common methodological foundations facilitate collaboration between biophysical scientists and economists, potentially resulting in more well-established collaborations between these disciplines (Mooney et al. 2013). Similarly, water quantity is likely the most commonly represented slow biophysical system process due to the relative ease of mathematical representation of hydrological dynamics. Simulating water quantity change requires relatively straightforward and highly robust algorithms that, for the most part, include well-known physical dynamics. However, simulating water quality requires describing complex, often non-linear, and less well-established algorithms, in part because biological processes are intimately involved.

## Model coupling methods

To reflect complex system dynamics, the development of coupled models requires highly interdisciplinary, collaborative teams. Incorporating multiple human-based disciplinary perspectives (e.g., social psychology *and* economics) in coupled models likely enables a more comprehensive representation of more types of human–freshwater feedback loops, including interactions between fast and slow processes within the human system. Importantly, each discipline needs to be included and contribute to the design of a project’s research questions, methods, and analytical approach. Whether and how this is done has been shown to make a difference in the quality of the interdisciplinary science that results (e.g., Cheruvelil et al. 2014).

All coupled human–freshwater system models we reviewed linked biophysical and economic variables. Biophysical models quantify environmental variables and economic models quantify the values people assign to goods and services, including market and non-market measures. Non-market values for ecosystem services may be quantified through estimates of stakeholders’ willingness-to-pay, measured in monetary units (e.g., estimation of how much a stakeholder is willing to pay for in-stream flow benefits through surveys, as in Fedra (2007). One significant challenge in establishing these linkages is that biophysical system models often operate on an hourly or daily time step, whereas economic models often operate on a seasonal or annual time step, requiring data (model output and input) aggregation or disaggregation techniques. For examples of specific data aggregation techniques, see Letcher et al. (2006), Jia et al. (2009), Roy et al. (2011), Daloğlu et al. (2014), Gandolfi et al. (2014), and Cabello et al. (2015). Every paper we reviewed simulated fast processes in biophysical and economic components, but only two simulated slow economic processes (Elshafei et al. 2014; Srinivasan 2015) and only one simulated a slow biophysical shift in environmental quality (van Delden et al. 2007).

Linking socio-cultural processes as part of coupled models often requires explicitly representing or accounting for held values: basic principles that shape how people assign value to specific environmental entities (Jones et al. 2016). The implementation of feedback loops with socio-cultural processes can be facilitated by eliciting the role of economic and socio-cultural factors in decision-making through participatory engagement of stakeholders with subsequent incorporation of those perspectives into the coupled model simulation (as in Fedra 2007). Alternatively, this can also be accomplished by simulating the role of social or cultural factors in determining the market value of ecosystem services. For example, Elshafei et al. (2014) simulated dynamic changes to “enviro-centrism” (a held value) as a result of changes in “community sensitivity,” or perceived threat to livelihood as a result of environmental change (context of valuation). In the model, dynamic community sensitivity has the capacity to alter the level of enviro-centrism, changing the way values are assigned to ecosystem services and thereby altering human decision-making (Elshafei et al. 2014).

Agent-based models (ABMs) that incorporate psychosocial, cognitive, or institution-based theories of human decision-making and behavior are particularly useful for representing socio-cultural processes of how held values factor into market processes and human decision-making (An 2012). Incorporating feedback loops with socio-cultural processes in coupled models can be challenging; however, explicit representation of held values, use of ABMs, and use of human behavior theories that include the role of social and cultural contexts for decision-making provide a path forward for incorporating both economic *and* socio-cultural processes in CNHS model feedback loops (An 2012; Jones et al. 2016; Schlüter et al. 2017).

## Novel insights with fast and slow feedback loops

Simulating fast and slow system processes together provides novel insights into complex system behavior. Time lags and the interaction of fast and slow processes in CNHS create non-linear complex system behavior (Filatova et al. 2016), in which a slow variable may remain relatively constant for years before changing, sometimes abruptly, in response to small but persistent forcing from fast variables. For example, changes in annual gross domestic product and water quality (fast variables) affect community sensitivity (a slow variable), which in turn affects human decision-making and subsequent gross domestic product and water quality (Elshafei et al. 2014). In this case, human decision-making in response to a given level of gross domestic product and water quality is context-dependent on the level of community sensitivity since people are more likely to adopt conservation measures when community sensitivity is high. Understanding how fast variables affect slow variables in this system is critical for predicting system trajectories: if the community sensitivity response to fast variables was not included in the coupled model, researchers may incorrectly assume that decision-making in response to a given gross domestic product and water quality will be the same today as in the future.

Additionally, since different stakeholders often have conflicting interests (e.g., agricultural development versus recreation), community sensitivity may differentially affect different stakeholder groups. In this case, the relative political power available to stakeholders is important in representing how decreasing water quality may in turn alter future local policy (Roy et al. 2011). Under conditions of high water quality, recreational stakeholders may not perceive threats to future recreation opportunities and thus may be relatively inactive in local policy debates. In contrast, under low water quality conditions, recreation-focused stakeholder groups may be more inclined to lobby for policy change that would improve water quality. Understanding the way slow human system processes—such as changes to policy or social norms—respond to fast biophysical and human system processes—such as changes in food production and water quality—is essential for informing potential long-term trajectories of CNHS. Our literature review emphasizes that a major challenge, and area for pushing knowledge forward in coupled human–natural systems, is to understand when and where changes in slow variables may occur as a result of changes in fast variables and how they control overall system dynamics.

Often, policy and management decisions are made in response to changes in fast variables without consideration of how the interaction of fast and slow variables determine overall system function and drive system dynamics over the long term. CNHS models that incorporate both fast and slow processes may reveal which management actions may be more effective over the long term. Currently, environmental decision-making often relies on “quick-fixes” (Stroh 2015) to initiate system change at “shallow” leverage points (Meadows 1999; Abson et al. 2017). Leverage points are focused alterations to a system (e.g., to a stock or flow, or to the system structure), which can initiate change in the rest of the system, and they range from least effective (shallow) to most effective (deep) at causing change to the overall system (Meadows 1999). Shallow leverage points are often interventions to fast variables and are the easiest to implement, but have the least potential to influence overall system dynamics (Abson et al. 2017). Shallow leverage points include policy changes to taxes, subsidies, and the rate of material flows in a system (e.g., regulating the extraction of water) (Abson et al. 2017). Since shallow leverage points do not necessarily account for fast–slow variable interactions, they often result in unintended consequences over the long term (Stroh 2015). An example of a shallow leverage point may be providing financial incentives to decrease the amount of phosphorus fertilizer applied on the land, which aims to affect the overall system by slowing the rate of phosphorus additions to the landscape. This management action is focused on controlling the rate of material flows in the system, but ignores the way that slow variables, such as social norms or community sensitivity, interact with fast variables to affect overall system dynamics, decreasing the effectiveness of the phosphorus incentive policy over the long term.

More effective, yet more difficult to implement leverage points are those that take into account an understanding of the system dynamics that arise out of the interaction between fast and slow variables, such as those that address system feedbacks (interaction between components), system design (social system drivers of feedbacks and parameters: e.g., power and structure of information flows), or system intent (system trajectory as a result of “values, goals, and world views of actors” in the system) (Abson et al. 2017). For example, rather than attempting to directly alter the rate of phosphorus fertilizer used through financial incentives as described above, a system design leverage point may alter information flows by reporting the quantity of phosphorus export from a land parcel directly to a landowner. Using a CNHS model to understand the processes of technology innovation or adoption as in Srinivasan (2015) elucidates a potential system design leverage point: altering the path of information flows of new technology may accelerate the rate of adoption. Altering the relative political power of stakeholder groups, as explored in Roy et al. (2011), is another potential system design leverage point. Using CNHS models with fast and slow variable interaction may increase system understanding among stakeholders, altering the most effective leverage points for system change: the intent of the system. Importantly, CNHS models that simulate both fast and slow human system processes foster new thinking around human–environment interactions and work toward whole-system understanding.

## Conclusions

There is a critical need for incorporating both fast and slow biophysical and human processes to simulate the feedback loops that fundamentally define CNHS over long time scales. Our literature review demonstrates that economics is often used to simulate fast human system processes over short time scales. The papers that simulated both economic and socio-cultural processes in feedback loops were more likely to also incorporate both fast and slow human system processes (two of the three papers). Thus, incorporation of both economic and socio-cultural factors in human decision-making may foster consideration of fast and slow interactions in CNHS (or vice versa).

Incorporating both fast and slow processes in coupled CNHS models enables exploration of time lags between slow and fast processes and threshold behavior in complex systems, working toward critical system understanding that fosters sustainable long-term decision-making. Improved understanding of complex dynamics has the potential to foster new understanding by researchers, decision-makers, and stakeholders, an essential step toward sustainable decision-making in complex systems (Matson et al. 2016). Incorporating both economic and socio-cultural processes in CNHS coupled models requires well-integrated interdisciplinary teams, use of aggregation techniques for linking economic and biophysical system models, and use of non-monetary ecosystem service valuations for linking socio-cultural and biophysical system processes (Cobourn et al. 2018).

Ultimately, incorporating fast and slow processes has the potential to reveal emergent system behaviors, such as how slow human system processes (social norms) may change as a result of biophysical variables (water quality and food production) and how these changes alter system trajectories. Incorporating fast and slow variables in CNHS models will also contribute to novel solution-oriented decisions, informing policies that focus on system feedbacks, system design, or system intent. Since the full dynamics of biophysical and human systems cannot be observed at any point in time or over the timeframe when many human decisions are made, modeling plays an important role in the future of human–environment research (Troy et al. 2015). We encourage adoption of CNHS models with integrated fast and slow processes to improve system representation and contribute to more sustainable decision-making.

## Electronic supplementary material

Below is the link to the electronic supplementary material.

Supplementary material 1 (PDF 48 kb)
