# Supplementary material for: Integrating fast and slow processes is essential for simulating human–freshwater interactions
Source: Ambio. 2018 Dec 19;48(10):1169–82. doi: 10.1007/s13280-018-1136-6 (PMC6722150; doi:10.1007/s13280-018-1136-6)
Supplement: Supplementary file 1 — Supplementary material 1 (PDF 48 kb) [file 13280_2018_1136_MOESM1_ESM.pdf]

Ambio

Electronic Supplementary Material

Title: **Integrating fast and slow processes is essential for simulating human—freshwater interactions**

Nicole K. Ward, Leah Fitchett, Julia A. Hart, Lele Shu, Jemma Stachelek, Weizhe Weng, Yu Zhang, Hilary Dugan, Amy Hetherington, Kevin Boyle, Cayelan C. Carey, Kelly M. Cobourn, Paul C. Hanson, Armen R. Kemanian, Michael G. Sorice, and Kathleen C. Weathers

Supporting Information

S 1. Abstract Screening Methods and Literature Keywords

We searched the literature using several citation indexing services with the keywords listed below, following the methods outlined in Figure 2. The Web of Science search resulted in 570 search results, SocIndex resulted in seven search results, and Water Resources Abstracts resulted in 50 search results. After removing duplicate papers, the initial search yielded 594 papers. We included relevant citations identified in references cited within the initial search results to yield a total of 601 papers. Keywords search was conducted on 15 August 2017.

We used the metagear R package (Lajeunesse, 2016) in the R statistical environment (R Core Team 2015) to distribute abstract screening effort among six co-authors of this manuscript with different disciplinary expertise. Each abstract was screened by two members of our research team. During screening, abstracts were marked as “yes,” “no,” or “maybe” as to if they fit our three qualifying criteria. The criteria used for the paper to be included in the review were that it had to: 1) be a watershed or freshwater-based study, 2) use at least one model representing the natural system and one model representing the human system, and 3) use a process-based model (mathematical equations representing physical, chemical, and biological processes) or empirical model (mathematical relationship of variables based on observed data) to represent the natural system, not a conceptual model (representation of system components without mathematical relationships).

Keywords:

(coupled OR linked OR integrated OR integrative) AND (model* OR simulation*) AND (“human-natural” OR “natural-human” OR “human natural” OR “natural human” OR “human

nature" OR "human-nature" OR "human-environment" OR "human environment" OR "social-ecological" OR "social ecological" OR "socio-ecological" OR "socio ecological" OR "social and ecological" OR "ecological and social" OR "socio-hydrology" OR "socio-hydrological" OR "integrated water resources management" OR "integrated water management" OR "integrated resource management" OR "integrated environmental modelling") AND (watershed OR "water resources" OR "water resource" OR "water management")

Sources:

Lajeunesse, M. J. 2016. Facilitating systematic reviews, data extraction and meta-analysis with the metagear package for R. Methods Ecol. Evol. **7**: 323–330. doi:10.1111/2041-210X.12472

R Core Team. 2015. R: a language and environment for statistical computing.
